# Supplementary material for: Effective surface conductivity of optical hyperbolic metasurfaces: from far-field characterization to surface wave analysis
Source: Sci Rep. 2018 Sep 20;8:14135. doi: 10.1038/s41598-018-32479-y (PMC6148295; doi:10.1038/s41598-018-32479-y)
Supplement: Supplementary file 1 — Supplementary Information [file 41598_2018_32479_MOESM1_ESM.pdf]

SUPPLEMENTARY INFORMATION

Effective surface conductivity of optical hyperbolic metasurfaces: from far-field characterization to surface wave analysis

Oleh. Y. Yermakov, Dmitry V. Permyakov, Filipp V. Porubaev, Pavel A. Dmitriev, Anton K. Samusev, Ivan V. Iorsh, Radu Malureanu, Andrei V. Lavrinenko, and Andrey A. Bogdanov

S1. Distribution of nanodisks sizes

The fabrication of a metasurface is still challenged and complicated technological process. Obviously, not all particles have identical parameters, i.e. there is a distribution of particles position and sizes. According to such a distribution shown in Fig. S1 we define the average values of the long and short axes of the elliptical nanodisks bases as $a_x = 134.06 \pm 10.22$ and $a_y = 103.05 \pm 4.54$ nm, respectively.

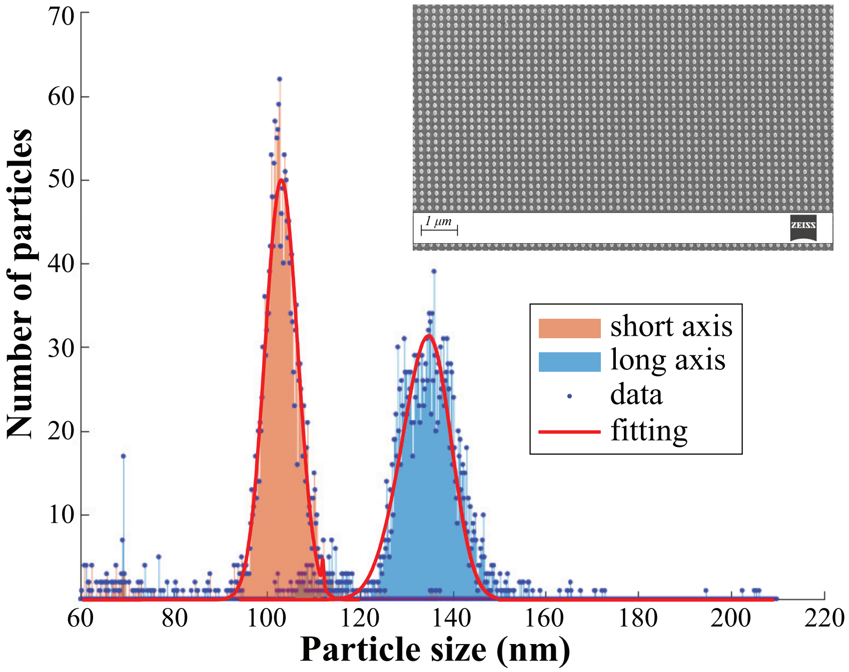

Figure S1. The distribution of the long and short axes of the elliptical nanodisks bases. The insert shows the SEM image of the fabricated metasurface sample.

S2. Polarizability of thin nanodisk with elliptical base

We define the polarizability of a nanodisk with elliptical base $\hat{\alpha}_0 = \mathrm{diag}(\alpha_x, \alpha_y, \alpha_z)$ through the polarizability of an ellipsoid. First, we consider the case of an ellipsoid with semiaxes b_x, b_y, b_z. For an anisotropic particle the depolarization factor N_i should be introduced[1]:

$$N_i = \frac{b_x b_y b_z}{2} \int_0^\infty \frac{ds}{\left(s+b_i^2\right)\sqrt{\left(s+b_x^2\right)\left(s+b_y^2\right)\left(s+b_z^2\right)}}, \qquad \text{(S1)}$$

$$i = x, y, z.$$

Three depolarization factors for any ellipsoid satisfy the relation: $N_x + N_y + N_z = 1$. Finally, the polarizability of the ellipsoid is

$$\alpha_i = \frac{b_x b_y b_z}{3} \frac{\varepsilon(\omega) - \varepsilon_m}{\varepsilon_m + N_i[\varepsilon(\omega) - \varepsilon_m]},\ i = x, y, z. \tag{S2}$$

Here ε_m is the permittivity of the surrounding medium and $\varepsilon(\omega)$ is the permittivity of the scatterer material. In case of a sphere, when $b_x = b_y = b_z$, the depolarization factor is $N_i = 1/3$ and we get the polarizability of a sphere according to Clausius-Mossotti relation[2].

Then, we switch from the ellipsoid to the elliptical nanodisk with sizes $a_x, a_y, a_z = H/2$ and make the substitution for the semiaxes $b_i = (1.5)^{1/3} a_i$, that takes into account the difference between the volumes of an ellipsoid and an elliptical cylinder. After that we use Eq. (S2) as the polarizability of the elliptical cylinder.

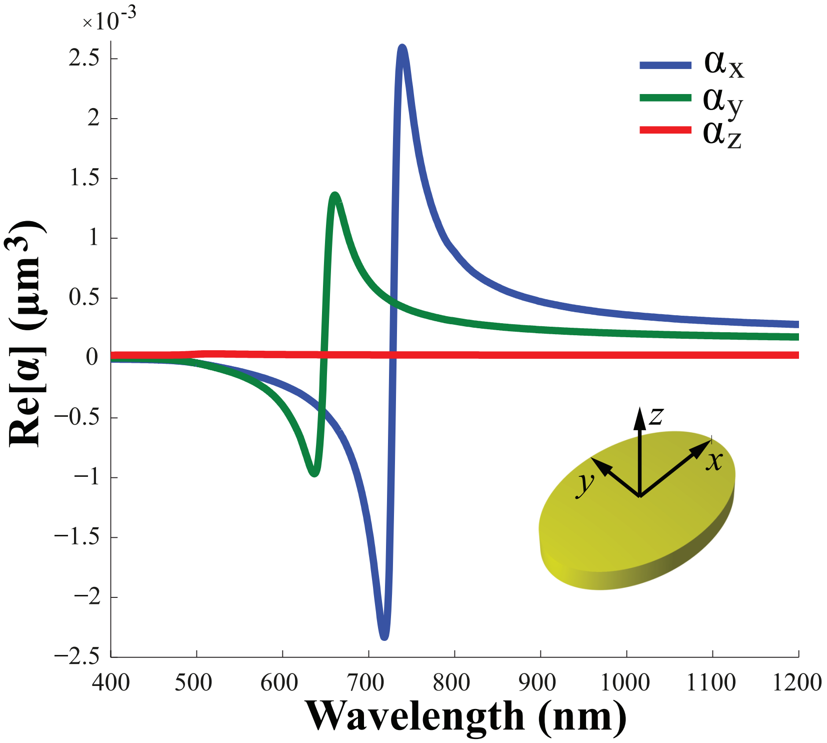

Figure S2. Real parts of the polarizability components for the thin nanodisk with elliptical base.

Thus, we obtain the polarizability of a thin nanodisk with elliptical base (Fig. S2). We conclude that resonance of the normal component of polarizability α_z is on very small wavelengths and for the studied range α_z is much smaller that in-plane components of polarizability. So, we can consider effective polarizability of thin nanodisk with elliptical base as a two-dimensional polarizability or conductivity tensor (5).

S3. Extraction of surface conductivity dispersion using fitting with Drude-Lorentz formula

We introduce the Drude-Lorentz model with three undefined coefficients β, γ, σ_0:

$$\sigma = \frac{i\sigma_0}{\beta + i\gamma}, \tag{S3}$$

where σ_0 is the amplitude of conductivity dispersion, $\beta = \omega - \Omega^2/\omega$, ω is the operating frequency, Ω is the spectral position of resonance, γ is the bandwidth of resonance.

Then we use Fresnel equations for a two-dimensional layer with an effective conductivity σ located between two isotropic media n_1 and n_2 in order to express the reflection coefficient:

$$r = \frac{n_1 - n_2 - \sigma}{n_1 + n_2 + \sigma}. \tag{S4}$$

Here we use Gauss units and express surface conductivity in the normalized dimensionless units $\sigma = 4\pi\widetilde{\sigma}/c$.

Substituting Eq. (S3) into Eq. (S4) we obtain

$$r = \frac{\beta(n_1 - n_2) + i[\gamma(n_1 - n_2) - \sigma_0]}{\beta(n_1 + n_2) + i[\gamma(n_1 + n_2) + \sigma_0]}, \tag{S5}$$

or expressing it through the reflectance

$$R = \frac{\sigma_0^2 + (\beta^2 + \gamma^2)(n_1 - n_2)^2 - 2\gamma\sigma_0(n_1 - n_2)}{\sigma_0^2 + (\beta^2 + \gamma^2)(n_1 + n_2)^2 + 2\gamma\sigma_0(n_1 + n_2)}. \tag{S6}$$

We consider a metasurface under consideration (Fig. 1) with the corresponding reflectance dispersion (Fig. 2a). Then we perform the fitting of the Eq. (S6) based on the least-squares method with Eq. (S3) [See Fig. S3a]. To achieve the same order of

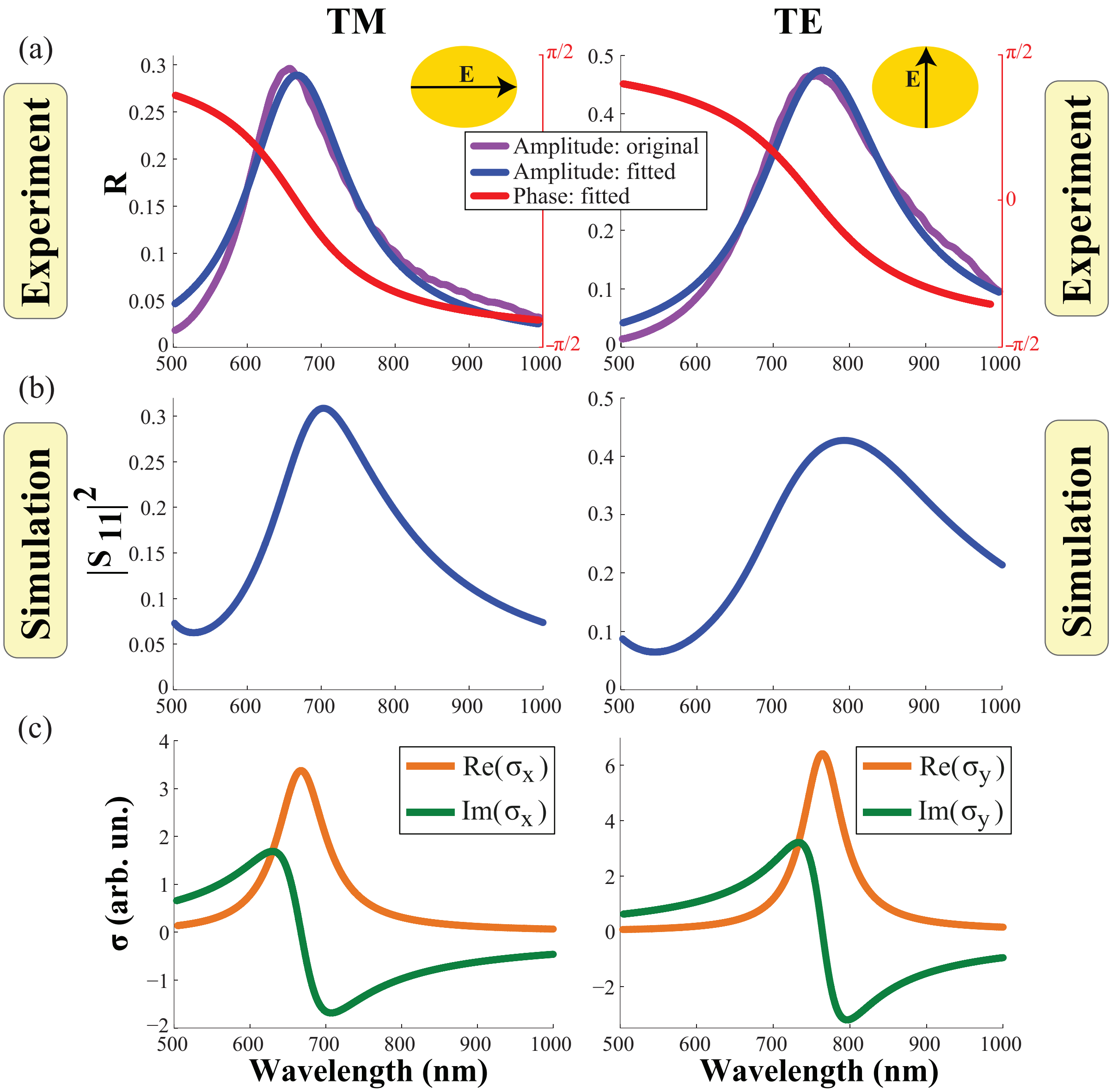

Figure S3. (a) Experimentally measured (purple) reflectance spectra of a metasurface for polarization along (left panel) and across (right panel) the long axis of the disk and its fitted amplitude (blue) and phase (red) by using Drude-Lorentz formula (S3). (b) The reflectance of a metasurface calculated in CST Microwave Studio with six times increased losses of the gold. (c) Real (orange lines) and imaginary (green lines) parts of the TM- and TE-polarized components of the effective surface conductivity tensor extracted from the fitted reflectance by using Eq. (S6).

the reflectance intensity in the simulation we increase the losses of the gold by six times (Fig. S3b). Hereinafter, we use the model of the gold permittivity from Ref. [3]. Finally, we obtain the dispersion of the surface conductivity for both polarizations from the fitting of the experimental data according to the Eq. (S3) [See Fig. S3c]. By knowing the parameters of fitting formula Eq. (S3) we can explicitly find the phase of the reflection coefficient from Eq. (S4) [See Fig. S3a].

S4. Performing surface conductivity extraction with oblique angle of incidence

The retrieval procedure of the effective surface conductivity within zero-thickness approximation can be applied for the arbitrary angle of incidence by modifying the Fresnel equations appropriately. Importantly, the final equations for the surface conductivity split by polarizations now and depend on the incident θ_i and refraction θ_t angles[4]:

$$\begin{aligned}\sigma^x &= \frac{n_1\cos\theta_t - n_2\cos\theta_i - S_{11}^x(n_1\cos\theta_t + n_2\cos\theta_i)}{\cos\theta_i\cos\theta_t(1+S_{11}^x)}\\ \sigma^y &= \frac{n_1\cos\theta_i - n_2\cos\theta_t - S_{11}^y(n_1\cos\theta_i - n_2\cos\theta_t)}{1+S_{11}^y}\end{aligned} \tag{S7}$$

These formulas are highly important in the case of the strong spatial dispersion, because they allow to determine the dependence of the retrieved conductivity on the in-plane wavevector.

S5. Phase correction in frame of zero-thickness approximation

Within zero-thickness approximation we substitute a plasmonic resonant metasurface of finite thickness H by a two-dimensional layer with effective conductivity σ. The obvious question arises: at which distance H_{eff} from the substrate we should dispose a two-dimensional layer? To define this distance we use a single criterion connected to the energy conservation law:

$$\mathrm{Re}(\sigma) > 0. \tag{S8}$$

The total phase of reflection coefficient (S-parameter) obtained by simulation in CST Microwave Studio is composed of two terms. The first one is an intrinsic phase associated directly with the reflection from a metasurface φ_0, while the second term is an extrinsic part caused by the electromagnetic waves propagation from the excitation port to metasurface and back (L_0 is a distance between port and top of a metasurface). It is extremely important to define the extrinsic phase and make the appropriate phase correction in order to obtain the reflection coefficient related to the metasurface properties intrinsically. So, we can express the total phase φ as

$$\varphi = \varphi_0 + k_0(2L_0 + H_{\text{eff}}), \tag{S9}$$

where $k_0 = n\omega/c$, n is a refractive index of the super- or substrate.

Figure S4 shows the real parts of conductivity tensor components for the different locations of a two-dimensional layer (top, middle and bottom of a metasurface of finite thickness). One can see that this criterion is satisfied only, when a two-dimensional layer is disposed at the distance $H/2$ from the substrate.

S6. Ewald summation for 2D square lattice

The lattice sums can be evaluated by using scalar Green's function in **r**-space and **k**-space:

$$\begin{aligned} g(r) &= \frac{e^{ikr}}{r} = -4\pi\int\frac{d^3k}{(2\pi)^3}\frac{e^{i\mathbf{kr}}}{k_0^2-k^2},\ r = |\mathbf{r}-\mathbf{r_i}|,\\ g_k(\mathbf{r},\mathbf{r_i}) &= \sum_i e^{i\mathbf{kr_i}}g(\mathbf{r}-\mathbf{r_i}).\end{aligned} \tag{S10}$$

Here k_0 is the wavevector of light in free space, $\mathbf{b}$ is the reciprocal wavevector of the structure, $\mathbf{r}$ is the excitation dipole or structural defect position and $\mathbf{r_i}$ is the position of i-th dipole of lattice structure. We consider the case $\mathbf{r} = (0,0,0)$.

Scalar Green's function (S10) can be divided into two parts by using Ewald summation with Ewald parameter K:

$$g_k(\mathbf{r}) = \sum_{\mathbf{b}} e^{i(\mathbf{k}+\mathbf{b})\mathbf{r}} f(\mathbf{k}+\mathbf{b}) + \sum_i e^{i\mathbf{kr_i}} F(|\mathbf{r}-\mathbf{r_i}|, K). \tag{S11}$$

For two-dimensional layer in the xy-plane with square lattice a the first term is defined through the function

$$f(\mathbf{k}+\mathbf{b}) = \frac{\pi}{a^2\kappa_{\mathbf{k}+\mathbf{b}}}e^{-\kappa_{\mathbf{k}+\mathbf{b}}|z|}\left[2-\mathrm{erf}\left(\frac{\kappa_{\mathbf{k}+\mathbf{b}}}{2K}-Kz\right)-\mathrm{erf}\left(\frac{\kappa_{\mathbf{k}+\mathbf{b}}}{2K}+Kz\right)\right], \tag{S12}$$

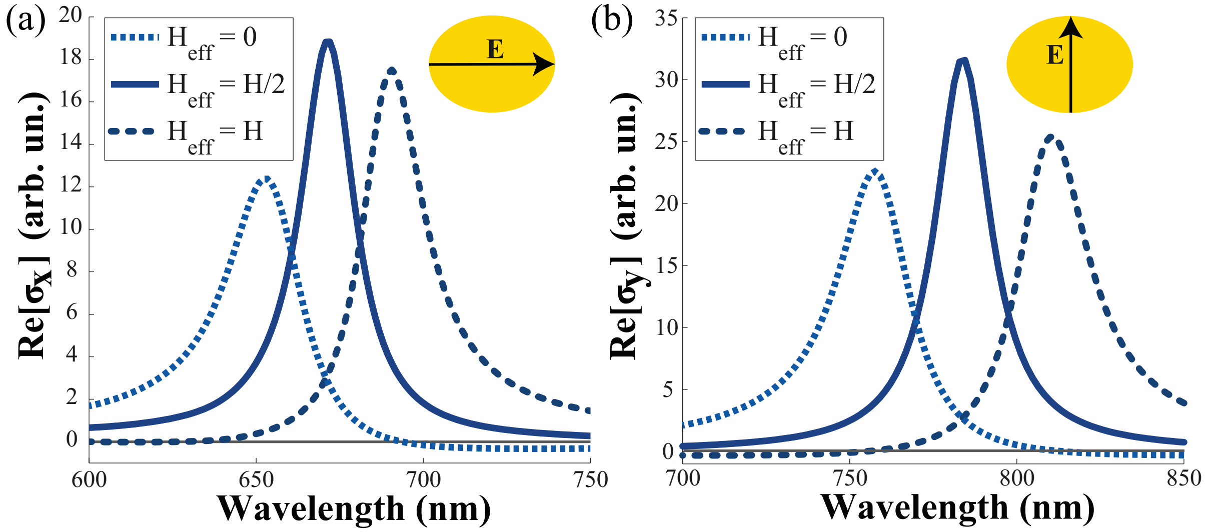

Figure S4. Real parts of conductivity for polarization along (a) and across (b) the long axis of the nanodisk at different distances between two-dimensional layer and substrate.

where

$$\kappa_{\mathbf{k}+\mathbf{b}} = \sqrt{(\mathbf{k}+\mathbf{b})^2 - k_0^2}, \tag{S13}$$

while the second term is expressed with the function

$$F(r,K) = \frac{\cos(k_0 r)}{r} - \left[\frac{e^{ik_0 r}}{2r}\mathrm{erf}\left(Kr + \frac{ik_0}{2K}\right) + \mathrm{c.c.}\right] \tag{S14}$$

The overall sum should be not significantly dependent on the Ewald parameter K. This parameter is taken as $K \sim 1/a$, where a is the lattice constant. So, we represent the Green's function as a sum of two contributions. The first term is calculated in real space, while the second is calculated in $\mathbf{k}$-space using Fourier transform. It significantly reduces calculation time, keeping accuracy up to 10^{-4}.[5]

S7. Impact of the difference between substrate and superstrate permittivities

The numerical and theoretical calculations have been performed under assumption of the homogeneous ambient medium. Indeed, the permittivities of the fused silica substrate and the immersion liquid may be slightly different in real experiment. Here, we analyze the impact of the perturbatively small difference between the permittivities of the substrate ε_0 and superstrate $\tilde{\varepsilon} = \varepsilon_0(1+\delta\varepsilon)$. Namely, we consider several cases of the permittivity difference from $\delta\varepsilon = -0.2$ to $\delta\varepsilon = 0.1$. The permittivity of substrate is chosen as the permittivity of the fused silica $\varepsilon_0 = 2.1$.

The simulation in CST Microwave Studio of the reflectance under normal incidence for both polarizations with different $\delta\varepsilon$ is shown in Figs. S5a,b. One can see that the bigger superstrate permittivity over substrate one leads to the redshift of the spectrum, while the blueshift takes place for the opposite situation. The dependences of the reflectance resonance position on the permittivity perturbation are presented in Fig. S5c for TM polarization and Fig. S5d for TE polarization. The dependence of the resonance position on the difference of the permittivities is close to linear. The exact coincidence of the resonances obtained experimentally and numerically has been achieved for $\delta\varepsilon = -0.2$ in TM polarization and $\delta\varepsilon = -0.16$ in TE polarization.

The modification of the discrete dipole model on the different substrate and superstrate for the surface conductivity tensor can be fulfilled by using the classical perturbation theory assuming $\delta\varepsilon \ll 1$. It results in the additional term in the conductivity tensor:

$$\delta\hat{\sigma}_{\mathrm{eff}} = -i\frac{4\pi\varepsilon_0\omega}{ca^2}\left(\delta\hat{\alpha}_{\mathrm{eff}} - \delta\varepsilon\hat{\alpha}_{\mathrm{eff}}\right). \tag{S15}$$

The correction factor of the permittivity perturbation for the effective surface polarizability $\delta\hat{\alpha}_{\mathrm{eff}}$ can be expressed through the corresponding perturbative terms for the disk polarizability and the interaction constant (as the substrate contribution to the

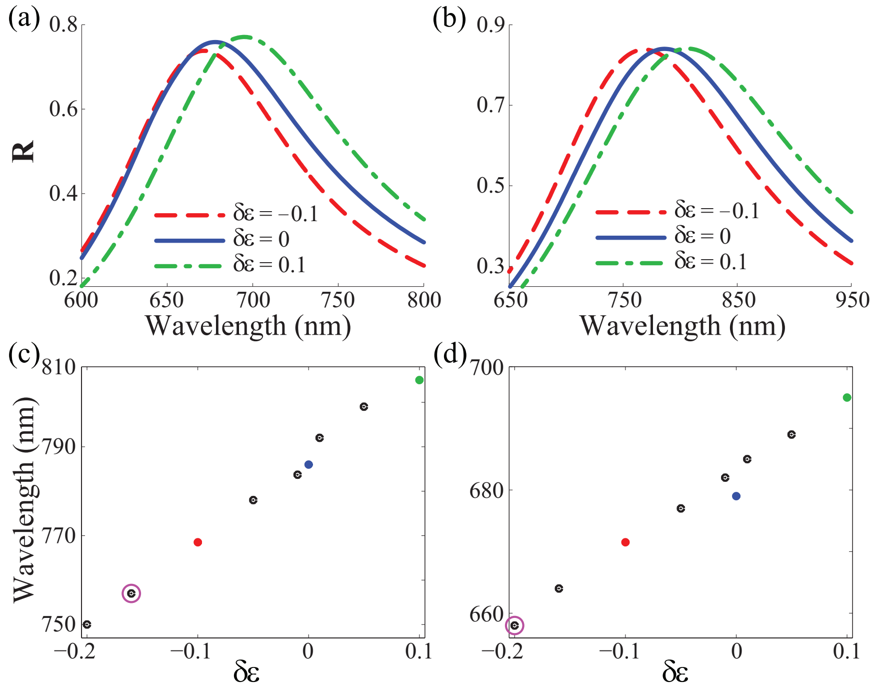

Figure S5. Reflectance spectra of a metasurface under normal incidence for TM (a) and TE (b) polarizations calculated numerically in CST Microwave Studio. The dependence of the resonance position wavelength on the permittivity perturbation $\delta\varepsilon$ for TM (c) and TE (d) polarizations. Magenta circles correspond to the resonances obtained experimentally.

Green's function) as follows:

$$
\begin{aligned}
&\delta\hat{\alpha}_{\text{eff}}^{-1} = \hat{\alpha}_0^{-1}\delta\hat{\alpha} - \delta\hat{G}.\\
&\delta\hat{\alpha} = \delta_{ik}\delta\alpha_i,\ i = x, y.\\
&\delta\alpha_i = \delta\varepsilon\frac{\varepsilon(\omega)}{[\varepsilon(\omega)-\varepsilon_0]\,[\varepsilon_0 + N_i(\varepsilon(\omega)-\varepsilon_0)]}.\\
&\delta\hat{G} = \delta\varepsilon\frac{2\pi k_0}{\varepsilon_0^2 a^2}\hat{I}.
\end{aligned}
\tag{S16}
$$

Here, $\hat{I}$ is the two-dimensional unit dyad, δ_{jk} is the Kronecker symbol.

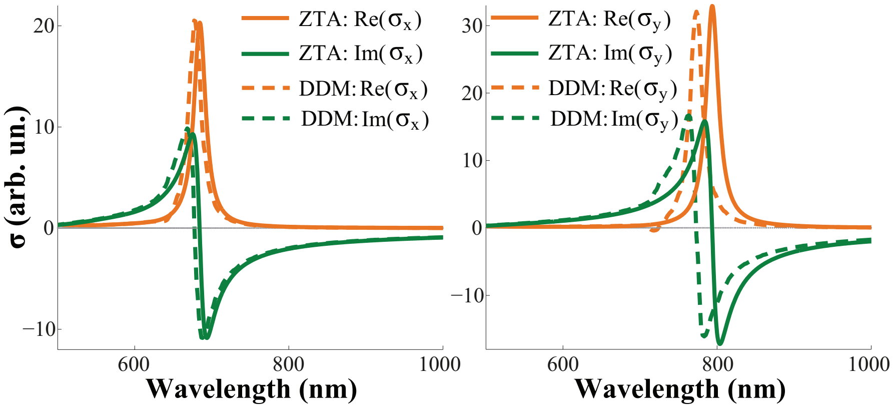

Figure S6. Real (orange lines) and imaginary (green lines) parts of the effective conductivity extracted via ZTA (solid lines) and DDM (dashed lines) in the case of difference between superstrate and substrate permittivities $\delta\varepsilon = 0.05$ for TM (a) and TE (b) polarizations.

Figure S6 shows the effective surface conductivity spectra for the fairly different substrate and superstrate materials ($\delta\varepsilon = 0.05$) calculated numerically and by using the DDM with perturbation corrections. One can see that the obtained results are in good agreement.

To conclude, we have shown that the effect of the difference between substrate and immersion liquid permittivities does not have a significant impact on the discussed results, but can lead to the better correspondence between the numerical calculations and the experimental measurements.

References

1. Landau, L. D. *et al. Electrodynamics of continuous media*, vol. 8 (Elsevier, 2013).

2. Jackson, J. D. *Classical electrodynamics* (John Wiley & Sons, 2007).

3. McPeak, K. M. *et al.* Plasmonic films can easily be better: rules and recipes. *ACS Photonics* **2**, 326–333 (2015).

4. Andryieuski, A. & Lavrinenko, A. V. Graphene metamaterials based tunable terahertz absorber: effective surface conductivity approach. *Opt. Express* **21**, 9144–9155 (2013).

5. Capolino, F., Wilton, D. R. & Johnson, W. A. Efficient computation of the 3D Green's function for the Helmholtz operator for a linear array of point sources using the Ewald method. *J. Comput. Phys.* **223**, 250–261 (2007).
